# The association between physical activity and mammography screening utilization: a longitudinal analysis, health retirement study (2004–2016)

**DOI:** 10.1186/s12889-025-23833-7

**Published:** 2025-09-02

**Authors:** Noof Alabdullatif, Alejandro Arrieta, Lucie Dlugasch, Nan Hu

**Affiliations:** 1 Department of Health Policy and Management, FIU Robert Stempel College of Public Health and Social Work, Miami, FL USA; 2 Department of Graduate Nursing, Nicole Wertheim College of Nursing & Health Sciences, Miami, FL USA; 3 Department of Biostatistics, FIU Robert Stempel College of Public Health and Social Work, 11200 8th Street, ACH5-467, Miami, FL 33199 USA

**Keywords:** Breast cancer screening, Mammography utilization, Physical activity, health disparity

## Abstract

**Purpose:**

Physical inactivity is a well-known factor associated with an increased risk of breast cancer. However, there is a disparity in physical activity levels among women in the United States. These disparities are associated with differences in women’s mammography screening behaviors, which may contribute to disparities in breast cancer incidence and outcomes. This study aims to evaluate the association between physical activity and the utilization of mammography screening. It also assesses whether this association is modified by women’s race/ethnicity and age.

**Methods:**

This is a longitudinal study that used the Health and Retirement Study data from 2004 to 2016. A total of 18,157 women aged 40 years and older were included. The 2004 wave was used as the baseline, with follow-up conducted in 2008, 2012, and 2016 (wave 9, 11, and 13 respectively). Mixed-effects logistic regression models were used, and odds ratios were reported.

**Results:**

The study found a significant positive association between physical activity and mammography utilization. After adjusting for confounding variables, women who were physically active had 1.31 times the odds of undergoing mammography screening compared to those who were inactive (95% CI: 1.13–1.51, *p* < 0.001). The association between physical activity and mammography screening utilization was weaker among Hispanic women.

**Conclusion:**

Interventions encouraging physical activity targeting racial/ethnic minorities may contribute to increasing mammography screening utilization and reducing breast cancer disparity.

**Supplementary Information:**

The online version contains supplementary material available at 10.1186/s12889-025-23833-7.

## Background

Breast cancer (BC) is a significant health issue globally and in the United States (U.S.) [1, 2]. Early detection through mammography screening is essential to improve BC prognosis and decrease BC mortality [1]. Physical inactivity is a well-established risk factor for BC, with research indicating that one-third of postmenopausal cases are attributable to modifiable factors such as physical inactivity [3]. Physical activity (PA) reduces BC incidence and mortality. In the U.S., the reduction in the risk of BC among active women compared to those who are inactive was estimated to be 10–20%, with greater risk reduction among those with higher intensity of activity [4–7]. Research has also shown that participating in moderate to vigorous PA reduces BC mortality, with greater mortality risk among those with lower intensity of activity [8, 9].

Engaging in PA has been associated with various health benefits, including a potential influence on preventive healthcare behaviors (e.g., mammography screening). Studies suggest that individuals who participate in PA may demonstrate a higher likelihood of engaging in other health-promoting behaviors, including adherence to preventive screenings [10]. Additionally, PA is associated with increased health literacy, which can lead to greater awareness about the importance of preventive measures such as cancer screening [11]. Active individuals may have better access to healthcare resources and information, facilitating timely mammography screenings [12]. Therefore, promoting PA could serve as a gateway to enhancing preventive healthcare behaviors, including mammography screening utilization. However, further research is needed to directly assess the association between PA and mammography screening utilization.

The Department of Health and Human Services’ Physical Activity Guidelines for Americans recommend adults do at least 150 to 300 min of moderate PA, or 75 to 150 min of vigorous aerobic PA per week, or an equivalent combination of both [13]. The Healthy People 2030 goals aim for 33.9% of adults to participate in at least moderate-intensity aerobic PA. However, recent data from 2020 shows that only 29.3% of adults were meeting this standard, falling short of the national target [14].

Despite the benefits of PA in reducing BC risk and mortality, approximately one in four adults 50 years and older are inactive [15]. According to the Centers for Disease Control and Prevention (CDC), the overall prevalence of physical inactivity in the U.S. was 25.3%^16^. The highest inactivity prevalence was among Hispanics (32%) followed by NH (non-Hispanic) Blacks (30%) and the lowest was among Asians (20.1%) followed by NH Whites (23%) [16]. Also, the prevalence of physical inactivity increases with increasing age among adults aged 50 years and older [15].

Given these disparities, it is important to understand whether PA is associated with mammography screening utilization and whether this relationship varies across racial/ethnic and age groups. This study addressed the following research question: Is women’s PA associated with mammography screening utilization? In this paper, the goal is to investigate the association rather than the causality. It was hypothesized that PA is positively associated with mammography screening utilization. This suggest that physically active women may be more exposed to health information, more aware of their health, and thus be more likely to seek preventive care such as screening compared to inactive women. This study also evaluated if this association is modified by women’s race/ethnicity and age.

## Methods

### Study population and sample

This is a longitudinal study that used data from the U.S. national Health and Retirement Study (HRS). The HRS is a longitudinal household survey launched in 1992 and conducted by the Institute for Social Research at the University of Michigan, with funding from the National Institute on Aging and the Social Security Administration. The HRS includes a nationally representative sample of older adults living in private households in the U.S. and collects extensive information on demographics, health status, health behaviors, health care utilization, employment, income, and wealth. Data are collected through telephone and in-person interviews, with follow-up surveys conducted every two years. New cohorts are enrolled every six years to maintain the study’s national representativeness over time. Further details of the HRS survey design can be found on the HRS website [17].

Although the (HRS) began in 1992 (wave 1), wave 7 (2004) was used as the baseline for this study because it was the first wave in which women were asked detailed questions about PA. A total of three follow-up time points were included in this analysis: waves 9, 11, and 13 (Years 2008, 2012, and 2016 respectively). Thus, wave 7 (2004) was used solely to measure PA, while mammography screening utilization was assessed beginning in Wave 9 (2008). HRS data file includes a total of 30,968 respondents. The study cohort includes women aged 40 years and older from the HRS data (*n* = 18157). Women aged 40 and older were included to align with United States Preventive Services Task Force (USPSTF) guidelines, which recommend individualized mammography screening for women aged 40–49 and routine biennial screening for women aged 50–74. Women under 40 and over 74 were excluded due to insufficient evidence regarding the benefits and harms of screening in these populations [18].

### Measures

In the HRS dataset, mammography screening utilization is assessed through self-reported responses. Beginning in wave 3 (1996), women were asked: “Did you have a mammogram or X-ray of the breast to search for cancer in the last two years?” In subsequent waves, the question was adapted to: “Did you have a mammogram or X-ray of the breast, to search for cancer since the previous wave?” This variable is binary, coded as 1 for “Yes” and 0 for “No.” The question was included in every other wave starting with waves 4 (1998) and 6 (2002), and asked of all respondents in odd-numbered waves beginning with wave 5 (2000), except for those who had already responded in the previous wave.

For this study, data from wave 7 (2004) were selected as the baseline, as it was the first wave to include detailed PA measures. Mammography utilization was assessed from wave 8 (2006) through wave 13 (2016)These time points were chosen to ensure sufficient follow-up and to align with the availability of consistent mammography data within the HRS.

The main exposure in this study is PA. From 2004 forward, three questions were asked about PA, each question represents a level of PA (e.g., vigorous activity, moderate activity, and light activity). Those questions are listed below and described more in HRS website [19].

- “How often do you take part in sports or activities that are vigorous, such as running or jogging, swimming, cycling, aerobics or gym workout, tennis, or digging with a spade or shovel?”
- “How often do you take part in sports or activities that are moderately energetic such as, gardening, cleaning the car, walking at a moderate pace, dancing, floor or stretching exercises?”
- “How often do you take part in sports or activities that are mildly energetic, such as vacuuming, laundry, home repairs?”

The questions use a five-point Likert scale with five possible response options: “every day; more than once per week; once per week; one to three times per month; or never”. A PA Index was created following the concept of a previously used method [20, 21]. Although guidelines often emphasize moderate and vigorous activity, light-intensity activity was included to provide a more comprehensive and realistic assessment of overall PA, particularly among older adults. Prior studies have also incorporated light activity, recognizing its potential contribution to health outcomes [22]. Light activity responses were coded as follows: 0 if women never engaged in PA, 1 if she engaged one to three times per month, 3 if she engaged once per week, 6 if she engaged more than once per week. Moderate activity responses were coded as follows: 0 if women never engaged in PA, 3 if she engaged one to three times per month, 6 if she engaged once per week, and 12 if she engaged more than once per week. Vigorous activity responses were coded as follows: 0 if women never engaged in PA, 6 if she engaged one to three times per month, 18 if she engaged once per week, 36 if she engaged more than once per week. The index was created by summing the scored responses to the light, moderate, and vigorous PA questions (possible range: 0–54). A binary variable was created to indicate PA in which women with a score lower than 21 were considered inactive. The cutoff value of 21 corresponds to the sample median PA index, providing a balanced division of participants by activity level. Additionally, we explored if PA has a delayed association with mammography screening utilization in the following year (is it a 1 year lag or a 1 period lag?). For that, we used a lag for PA (PA from the one year before the current measurement). A lagged PA was used as the main exposure in this analysis to ensure that the exposure occurred before the outcome, thereby enhancing the temporal structure of the analysis.

Many covariates were adjusted for in the full model, including women’s demographic, socioeconomic, and health-related variables. Age was included as a continuous variable, calculated as: (interview date– respondent’s birth date)/365.25. Other demographic variables include race/ethnicity which was categorized into three groups “non-Hispanic White; non-Hispanic Black; and Hispanic”, marital status, defined as a binary variable “currently married and otherwise”, and regions, grouped into four groups “Northeast; Midwest; South; and West”. Health insurance coverage was included as a binary variable “insured and uninsured”. Socioeconomic variables include education level, categorized as “less than high school; high school/some college; and college degree or more” and household income, classified into five categories “$0 - $10,000; $10,000 - $18,000; $18,000 - $29,000; $29,000 - $49,000; and $49,000+”. Health status was adjusted using five self-reported categories “poor; fair; good; very good; and excellent”. The model also included adjustment for the year of the survey, covering the four waves “2004; 2008; 2012; and 2016”.

### Statistical analysis

Data were summarized as frequency (N) and percentage (%) for categorical variables and mean and standard deviation (SD) for continuous variables. Several models were tested in this analysis to investigate the association between PA and mammography screening utilization, including separate models to assess potential effect modifications by race/ethnicity and age. The association between PA and mammography screening utilization was assessed using the mixed-effects logistic regression model [23]. Odds ratios (OR) were reported to measure the odds of utilizing mammography screening among active women compared to inactive women. The equation for the mixed-effects logistic regression model can be expressed as:

1 $$logit\mathit\;\mathit{\left[{\mathrm P\mathrm{\left({Y_{\mathrm{ij}}=1}\right)}}\right]}\mathit\;=\beta_0\;+\;{\sum\nolimits^{p}_{k=1}}\;{\mathrm\beta}_{\mathrm i}{\mathrm X}_{\mathrm{ijk}}$$

where *i* (*i* = 1,…,n) is the index of study participants in the database (*n* = 18157), *j* is the index of years since 1996 (*j* = 0,…,6), k is the index of independent variables (k = 1, 2,…p; and p is the total number of independent variables in the model). Y_ij_ denotes mammography screening utilization for participant *i* at time j. Y_ij_ is a binary variable in which Y_ij_=0 indicates that women didn’t use mammography screening while Y_ij_=1 indicates that women used mammography screening. X_ij_ denotes the vector of independent variables for participant *i* at time *j.*

To assess the effect modification by race/ethnicity in the relationship between the PA and mammography screening utilization, race/ethnicity “Hispanic; Non-Hispanic (NH) White; and NH Black” was used as an effect modifier in the model with specifying “NH White” as the reference. Missing observations (0.15%) were treated using complete case analysis. Stata (Stata Corp., College Station, TX, USA) version 16 was used to conduct the analysis. Results with *p*-value < 0.05 are considered statistically significant. Strengthening the Reporting of Observational Studies in Epidemiology Statement checklist (STROBE) was followed [24]. IRB approval was not required for this study due to using a publicly accessible national data source (HRS).

## Results

**Fig. 1:**
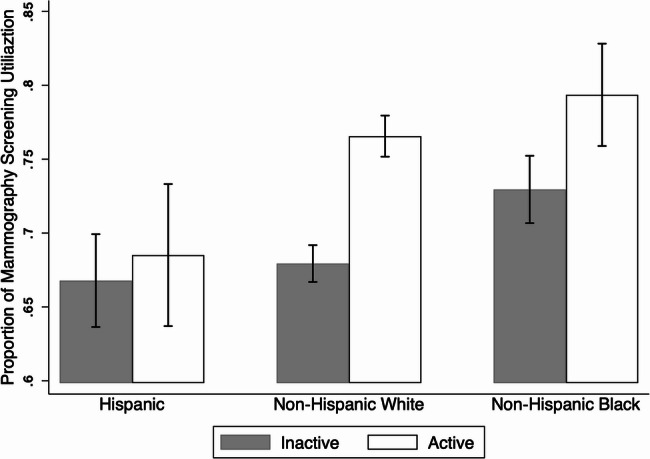
Sample estimate of proportion of mammography screening utilization between active and inactive women across different races/ethnicities

Table 1 shows the demographic and socioeconomic factors of women aged 40 years and older. The average age of the total women in the sample was 70.36 years. Approximately 73% of the women in this sample were NH Whites, and around half of them were married (52.32%). Most women in the sample were insured (96.01%) and about 27% of them were from the lowest household income quantile ($0- $10,000). 63% of women were inactive. Participants’ characteristics were also presented by PA status to examine potential baseline differences between active and inactive individuals (Table S1). Physically active individuals were more likely to be younger (particularly in the 40–49 age group), Hispanic, unmarried, and have higher levels of education and income compared to those who were inactive (all *p* < 0.01). Regional differences were also noted, with individuals in the Western United States being less active than those in other regions (*p* < 0.001). Health status showed a clear gradient, with better self-reported health associated with higher physical activity levels (*p* < 0.001). However, there were no significant differences in PA levels by insurance status (*p* = 0.844). Figure 1. showed that across all ethnicities, active women were more likely to utilize mammography screening compared to inactive women.

**Table 1:** Characteristics of study cohort among female respondents aged 40+, 2004–2016 (*n* = 18157)

Variable	Mean (SD)	Median	Min	Max
Age (Continuous)	70.36 (7.43)	70.83	40	97

Variable	Frequency (N), Percentage (%)			
Age category				
40- 49	170 (0.94)			
50-59	995 (5.48)			
60-75	11717 (64.55)			
75+	5257 (29.06)			
Race/ethnicity				
NH White^a^	12994 (73.31)			
NH Black^b^	2951 (16.65)			
Hispanic	1780 (10.04)			
Marital status				
Unmarried	8657 (47.68)			
Married	9500 (52.32)			
Insurance coverage				
Uninsured	725 (3.99)			
Insured	17432 (96.01)			
Region				
Northeast	2728 (15.04)			
Midwest	4239 (23.37)			
South	7967 (43.93)			
West	3129 (17.25)			
Household income quantiles				
$0 - $10000	4721 (26.90)			
$10000 - $18000	3660 (20.85)			
$18000 - $29000	3276 (18.66)			
$29000 - $49000	3064 (17.46)			
$49000+	2831 (16.13)			
Education				
Less than high school	4142 (22.82)			
High school/some college	11122 (61.27)			
College and above	2888 (15.91)			
Health status				
Excellent	868 (8.83)			
Very good	2002 (20.36)			
Good	3340 (33.97)			
Fair	2761 (28.08)			
Poor	862 (8.77)			
PA^c^				
Inactive	7932 (63.40)			
Active	4579 (36.60)			
Survey year				
2004	5288 (29.12)			
2008	4918 (27.09)			
2012	4403 (24.25)			
2016	3548 (19.54)			

^a^NH White: Non-Hispanic White

^b^NH Black: Non-Hispanic Black

^c^PA: physical activity based on the PA Index, a score of 21 and above indicates active whereas a score lower than 21 indicates inactive

This study examined the relationship between PA and mammography screening utilization among women aged 40 years and above (*n* = 18157, Table 2). Unadjusted and adjusted models showed a significant relationship (*p* < 0.001 for both models) between women’s PA and their mammography screening utilization in the following year. Based on the adjusted model, physically active women had 1.31 times the odds of utilizing mammography screening compared to inactive women (OR: 1.31, 95% CI: 1.13–1.51).

**Table 2:** ORs and 95% CIs for association between PA ^a^ and mammography screening utilization (*n* = 18157)

Mammography screening utilization	Adjusted OR (95% Conf. Interval)	*P*	Unadjusted OR (95% Conf. Interval)	*P*
PA^a^				
Inactive	Reference			
Active	1.31 (1.13, 1.51)	<0.001	1.52 (1.33, 1.74)	<0.001
Age	0.96 (0.95, 0.98)			
Education				
Less than high school	Reference			
High school/some college	1.23 (0.98, 1.56)			
College and above	1.62 (1.17, 2.24)	0.003		
Household income quintiles				
$0 - $10,000	Reference			
$10,000 - $18,000	1.34 (1.10, 1.64)	0.003		
$18,000 - $29,000	1.67 (1.33, 2.09)	<0.001		
$29,000 - $49,000	2.02 (1.58, 2.58)	<0.001		
$49,000+	2.29 (1.75, 3.02)	<0.001		
Race/ethnicity				
NH White^b^	Reference			
NH Black^c^	2.42 (1.88, 3.12)	<0.001		
Hispanic	1.74 (1.26, 2.39)	0.001		
Health insurance				
Uninsured	Reference			
Insured	5.48 (3.57, 8.29)	<0.001		
Region				
Northeast	Reference			
Midwest	1.01 (0.75,1.34)	0.966		
South	0.95 (0.73, 1.23)	0.711		
West	0.89 (0.65, 1.22)	0.482		
Marital status				
Not married	Reference			
Married	1.94 (1.63, 2.29)	<0.001		
Health status				
Poor	Reference			
Fair	0.40 (0.27, 0.57)	<0.001		
Good	0.71 (0.52, 0.97)	0.034		
Very good	0.87 (0.64, 1.16)	0.347		
Excellent	1.21 (0.91, 1.62)	0.187		
Survey year				
Year 2004	Reference			
Year 2008	0.98 (0.87, 1.11)	0.795		
Year 2012	0.63 (0.56, 0.71)	<0.001		
Year 2016	0.38 (0.33, 0.43)	<0.001		

^a^PA: physical activity based on the PA index, a score of 21 and above indicates active whereas a score lower than 21 indicates inactive

^b^NH White: Non-Hispanic White

^c^NH Black: Non-Hispanic Black

The difference among race/ethnicity in the relationship between PA and mammography screening utilization was assessed (Table 3). Hispanic women showed the weakest association between PA and mammography screening compared to all other racial/ethnic groups. The impact of PA on mammography screening utilization was significantly lower among Hispanic women than NH White women (95% CI: 0.40–0.96, *p* = 0.032). However, the study found that categorical age “40–49; 50–59; and 60–75” was not an effect modifier in the relationship between PA and mammography screening utilization (Table S2).

**Table 3:** Coefficients and 95% CIs; effect modified by race/ethnicity (*n*= 18157)

Mammography screening utilization	Coefficient (95% Conf. Interval)	*P*
PA^a^	Reference	
Inactive		
Active	1.68 (1.44, 1.96)	<0.001
Race/ethnicity	Reference	
NH White^b^		
Hispanic	0.90 (0.67, 1.22)	0.503
NH Black^c^	1.47 (1.14, 1.88)	0.003
Effect modification by race/ethnicity	Reference	
NH White^b^		
Hispanic	0.62 (0.40, 0.96)	0.032
Active vs Inactive	0.86 (0.58, 1.29)	0.472
NH Black^c^		
Active vs Inactive		

^a^PA: physical activity based on the PA Index, a score of 21 and above indicates active whereas a score lower than 21 indicates inactive

^b^NH White: Non-Hispanic White

^c^NH Black: Non-Hispanic Black

## Discussion

This research is among the first longitudinal studies to evaluate the association between PA and utilization of mammography screening in the U.S. The results suggest that PA is positively associated with mammography screening utilization. The study showed that active women in the U.S. were more likely to utilize mammography screening compared to inactive women, and the association was the weakest among Hispanics. Based on the unadjusted model, physically active women had 1.52 times the odds of undergoing mammography screening compared to inactive women (95% CI: 1.33–1.74, *p* < 0.001). After adjusting for women’s variables, the odds decreased to 1.31 (95% CI: 1.13, 1.51, *p* < 0.001). Also, the analysis showed a growth in the proportion of mammography screening utilization between active and inactive women from 2008 to 2012 (Figure S1).

The findings of this study should not be interpreted as evidence of a direct causal relationship between PA and mammography use. Rather, the observed association may reflect a broader pattern of health-conscious behaviors, where individuals who engage in regular PA are also more likely to participate in preventive health measures such as screening. In this context, PA may act as an indicator of general health engagement rather than a direct impact on the screening utilization.

The main exposure of this longitudinal study is PA measurement immediately before the current measurement time. The lagged PA was used rather than the current PA because it will better reflect the causal relationship. Specifically, a period of time is needed for the impact of PA to influence the utilization of mammography screening after engagement. However, similar results were found when the association between current PA and mammography screening utilization was assessed. Physically active women had 1.30 times the odds of utilizing mammography screening compared to inactive women (95% CI: 1.16–1.48, *p* < 0.001, Table S3).

Our results support the theory that physically active women are more likely to encounter health-related information and have greater awareness of their well-being, which may lead to a higher likelihood of engaging in mammography screening compared to inactive women. Some studies in Australia found similar results that women who were more physically active were more likely to have ever had a mammogram [25, 26]. Lagerlund et al. (2015) also found that nonattendance at a population-based invitational mammography screening program in Southern Sweden was associated with many lifestyle factors including physical inactivity [27]. Another Brazilian study found that inadequate mammography screening compared to the recommended timespan by the Ministry of Health was found to be associated with physical inactivity [28]. Furthermore, a national study that focused on Korean American women found that women who walked at least 10 min per week were 61 times more likely to have ever had a mammogram [29].

To the best of our knowledge, this work is the first study to analyze the effect modification by race/ethnicity in the relationship between PA and mammography screening utilization (Table 3). The association didn’t significantly vary between NH Black and NH White women. Conversely, the association of PA on the utilization of mammography screening was significantly lower among Hispanic women compared to NH Whites. The effect modification by race/ethnicity was conducted using both unadjusted and fully adjusted models. While the unadjusted models suggested potential differences in the association between PA and the mammography screening across racial/ethnic groups, these interactions became non-significant after adjusting for covariates such as education, income, and health status. One possible explanation for this is that many of these covariates are strongly correlated with race/ethnicity, which may have introduced multicollinearity, so it may impact the detection of effect modification. Therefore, our conclusions are based on the unadjusted models, which more clearly reflect the observed patterns and potential disparities.

A stratified analysis was also conducted and showed similar conclusions to the effect modification results. A significant association was found between PA and mammography screening utilization among non-Hispanic White women (*P* = 0.002, Table S4); however, the association was not statistically significant among non-Hispanic Black and Hispanic women (Table S5 and Table S6 *P*≥ 0.059). While NH White women served as the reference group in our interaction models, the relatively smaller association observed among Hispanic women warrants attention. This weaker association may be influenced by factors such as cultural attitudes toward preventive care, differences in access to healthcare services, or variations in health literacy. These findings highlight the importance of considering sociocultural and systemic factors when promoting screening behaviors across diverse populations. Racial/ethnic minority groups, such as Hispanic and NH Black women, may share some socioeconomic and health behavior patterns due to structural and systemic factors; however, important differences and diversity exist within and between these groups. Yaghjyan et al. found that NH Black and Hispanic BC survivors were more likely to engage in unhealthy behaviors including physical inactivity compared to NH Whites [30]. Thus, it is suggested that the non-significant difference between NH Black and NH Whites in this association might be due to the small proportion of NH Black women in the sample (16.65%, Table 1). Additionally, analysis of effect modification using current PA showed significant associations with mammography screening across all racial/ethnic groups, with the weakest association observed among Hispanic women (*p* $\:\le\:$ 0.003, Table S7).

For race/ethnicity as a control variable in the adjusted model, compared to NH White women, both NH Black and Hispanic women showed a significant decrease in mammography screening utilization (*p* $\:\le\:$ 0.001, Table 2). Also, Fig. 1, supported the main finding that PA increased the odds of utilizing mammography screening among women from all races/ethnicities. For active women, the highest proportion of mammography screening utilization was found among NH Black women followed by NH White women. Similarly, for inactive women, the highest proportion of mammography screening utilization was found among NH Black women followed by NH White women. The lowest utilization proportions were observed among Hispanic women for both active and inactive women.

In this sample, NH Black and Hispanic women aged 40 years and above were different in many socioeconomic factors. Disparities in those factors may contribute to the disparity in lifestyle behavior and consequently in the utilization of mammography screening. For instance, 67% of NH Whites had at least a high school degree and 18% of them had a college degree or above, however among Hispanic women, 63% of them had less than high school degree (Table S8). Similarly, only 18% of NH White women were from the lowest household income quantile ($0 - $10,000) compared to 58% of Hispanic and 47% of NH Black women (Table S9).

The factors that can influence the health and health behaviors of an individual could be shaped by the structural, economic, and social inequities between NH Whites and racial minorities [1]. Socioeconomic differences (e.g., income and education) between NH Whites and racial minorities (NH Black and Hispanic) are also an important factor in the disparity in physical inactivity [31]. Although participating in PA improved over time across all racial/ethnic groups, the disparity in PA between Whites and racial minorities narrowed but persisted between them and across all income levels [31]. Also, experiencing discrimination and racism might have an impact on women’s healthy lifestyle behavior such as PA, and thus contribute to the health disparity in mammography screening utilization. For instance, neighborhood poverties were found to be associated with increased odds of physical inactivity among both Whites and Blacks [32]. Wilson-Frederick et al. (2014) have assessed whether racial/ethnic disparities in physical inactivity are present among Blacks and Whites living in similar social contexts and found that Blacks still had higher odds of inactivity compared to Whites [33]. Other cultural or mental health factors such as stress could play a role in the disparity of health behavior between Whites and other racial/ethnic minorities [34].

The findings of this study have important public health implications, particularly in addressing health disparities in mammography screening utilization. The positive association between PA and increased mammography screening utilization suggests that encouraging PA could be an effective strategy to improve screening rates, especially among women who are at higher risk for BC. Public health initiatives should focus on promoting PA as part of broader cancer prevention strategies, emphasizing its role in improving women’s health behaviors. Eliminating racial/ethnic disparities in PA requires addressing the associated barriers with targeting underserve d populations such as providing safe neighborhoods, parks, and sidewalks [16]. Tailored interventions to educate women about the importance of limiting sedentary behaviors would help to eliminate health disparity [1]. By enhancing access to supportive environments for PA, policymakers can help reduce barriers to both PA and mammography screening.

Future research should aim to better understand the underlying causes of these disparities and assess the effectiveness of interventions designed to promote both PA and mammography screening among underserved populations. Additionally, BC prevention research should prioritize racial/ethnic minorities and underserved groups to advance health equity among women in the U.S.

This study provides externally valid results using HRS; the largest national database that investigates work, aging, and retirement in the U.S. The longitudinal design allowed for observing patterns of the association between PA and mammography screening utilization from one year to another. However, this study has some limitations. First, the study is prone to social desirability bias because it used self-reported outcome, exposure, and confounding variables. Second, this analysis assesses the association, and causality couldn’t be established based on the results. Last, the analysis was limited to what is available in the dataset. For instance, variables that describe the actual intensity of PA are not available (e.g., how much time is spent in PA).

## Conclusions

To our knowledge, this is one of the few studies that investigate the relationship between PA and mammography screening utilization in the U.S. A strong positive association was found, which varied by women’s race/ethnicity, with Hispanic women showing the weakest association between PA and mammography screening. The present results contribute to the evidence that PA has a positive impact on women’s health and women’s health-related behaviors. Communities must be designed to provide a safe environment and interventions that encourage PA equitably for all people from different races/ethnicities in the U.S.

## Supplementary Information

Supplementary Material 1.

## Data Availability

The datasets generated and/or analyzed during the current study are available in the HRS repository, https://hrsdata.isr.umich.edu/data-products/rand.

## Abbreviations

BC: breast cancer
PA: physical activity
NH: non-Hispanic
HRS: Health and Retirement Study
OR: Odds ratio
