# Supplementary material for: The association between physical activity and mammography screening utilization: a longitudinal analysis, health retirement study (2004–2016)
Source: BMC Public Health. 2025 Sep 2;25:3032. doi: 10.1186/s12889-025-23833-7 (PMC12406420; doi:10.1186/s12889-025-23833-7)
Supplement: Supplementary file 1 — Supplementary Material 1. [file 12889_2025_23833_MOESM1_ESM.docx]

**Supplementary Materials**

**1. Characteristics of Participants**

Table S1. Baseline characteristics of the study population by physical activity status (percentages)

| **Variable** | **Active** | **Not active** | ***P*** |
| --- | --- | --- | --- |
| Age Category  40- 49  50-59  60-75  75+ | 65.52  58.57  62.42  65.16 | 34.48  41.43  37.58  34.84 | 0.003 |
| Race/ethnicity  NH White ^a^  NH Black ^b^  Hispanic | 70.56  60.11  73.57 | 29.44  39.89  26.43 | <0.001 |
| Marital Status  Unmarried  Married | 68.16  58.47 | 31.84  41.53 | <0.001 |
| Education  Less than high school  High school/ some college  College and above | 74.26  62.66  51.40 | 25.74  37.34  48.60 | <0.001 |
| Insurance Coverage  Uninsured  Insured | 62.90  63.41 | 37.10  36.59 | 0.844 |
| Region  Northeast  Midwest  South  West | 65.33  63.06  64.94  58.43 | 34.67  36.94  35.06  41.57 | <0.001 |
| Household Income Quantiles  $0 - $10000  $10000 - $18000  $18000 - $29000  $29000 - $49000  $49000+ | 72.86  66.29  61.77  55.05  51.69 | 27.14  33.71  38.23  44.95  48.31 | <0.001 |
| Health Status  Excellent  Very good  Good  Fair  Poor | 40.96  53.23  64.81  75.09  80.98 | 59.04  46.77  35.19  24.91  19.02 | <0.001 |
| Survey Year  2004  2008  2012  2016 | 65.35  62.73  61.62 | 34.65  37.27  38.38 | 0.001 |

**2. Effect modification by age on the association between PA and mammography screening utilization**

| Table S2. Coefficients (log of odds ratio) and 95% confidence intervals from mixed effect logistic regressions of MS utilization based on PA; effect modified by age | | |
| --- | --- | --- |
| Mammography Utilization | Coefficient  (95% Conf. Interval) | *P* |
| PA ^a^  Not active  Active  Age  Age 40-49  Age 50-59  Age 60-75  Effect modification by age  Age 50-59  Active vs not active  Age 60-75  Active vs not active | Reference | |
|  | 10.81 (1.24, 94.12) | 0.031 |
|  | Reference | |
|  | 1.44 (0.44, 4.69) | <0.001 |
|  | 2.39 (0.76, 7.52) | <0.001 |
|  | 0.15 (0.01, 1.44) | 0.102 |
|  | 0.15 (0.02, 1.29) | 0.084 |
| ^a^ PA: physical activity based on the PA Index, a score of 21 and above indicates active whereas a score lower than 21 indicates inactive. | | |

**3. Association between physical activity PA and mammography screening utilization**

| Table S3. Odds ratios and 95% confidence intervals from mixed effect logistic regressions of mammography screening based on PA | | | | | | | | | | | |
| --- | --- | --- | --- | --- | --- | --- | --- | --- | --- | --- | --- |
| Variable | | Odds Ratio | | *P* | 95% Conf. Interval | | Odds Ratio | *P* | | 95% Conf. Interval | |
| PA ^a^ | | Current PA | | | | | Lag PA | | | | |
|  | Not active | Reference | | | | | Reference | | | | |
|  | Active | 1.30 | <0.001 | | 1.16 | 1.48 | 1.31 | <0.001 | | 1.13 | 1.51 |
| Age | | 0.97 | <0.001 | | 0.96 | 0.99 | 0.96 | <0.001 | | 0.95 | 0.98 |
| Education | |  |  |  |  |  |  | | | | |
|  | Less than high school | Reference | | | | | Reference | | | | |
|  | High school/some college | 1.35 | 0.003 | | 1.11 | 1.64 | 1.23 | 0.072 | | 0.98 | 1.56 |
|  | College and above | 1.89 | <0.001 | | 1.43 | 2.50 | 1.62 | 0.003 | | 1.17 | 2.24 |
| Household income quintiles | |  |  |  |  |  |  | | | | |
|  | $0 - $10,000 | Reference | | | | | Reference | | | | |
|  | $10,000 - $18,000 | 1.29 | 0.001 | | 1.10 | 1.51 | 1.34 | | 0.003 | 1.10 | 1.64 |
|  | $18,000 - $29,000 | 1.49 | <0.001 | | 1.25 | 1.79 | 1.67 | | <0.001 | 1.33 | 2.09 |
|  | $29,000 - $49,000 | 1.80 | <0.001 | | 1.47 | 2.18 | 2.02 | | <0.001 | 1.58 | 2.58 |
|  | $49,000+ | 2.21 | <0.001 | | 1.77 | 2.76 | 2.29 | | <0.001 | 1.75 | 3.02 |
| Race/ethnicity | |  |  |  |  |  |  | | | | |
|  | Non-Hispanic White | Reference | | | | | Reference | | | | |
|  | Hispanic | 1.81 | <0.001 | | 1.37 | 2.38 | 1.74 | 0.001 | | 1.26 | 2.39 |
|  | Non-Hispanic Black | 2.00 | <0.001 | | 1.61 | 2.48 | 2.42 | <0.001 | | 1.88 | 3.12 |
| Health insurance | |  |  |  |  |  |  | | | | |
|  | Uninsured | Reference | | | | | Reference | | | | |
|  | Insured | 4.63 | <0.001 | | 3.52 | 6.09 | 5.48 | | <0.001 | 3.57 | 8.29 |
| Region | |  |  |  |  |  |  | | | | |
|  | Northeast | Reference | | | | | Reference | | | | |
|  | Midwest | 0.94 | 0.638 | | 0.74 | 1.20 | 1.01 | 0.966 | | 0.75 | 1.34 |
|  | South | 0.97 | 0.849 | | 0.79 | 1.22 | 0.95 | 0.711 | | 0.73 | 1.23 |
|  | West | 0.91 | 0.510 | | 0.70 | 1.20 | 0.89 | 0.482 | | 0.65 | 1.22 |
| Marital status | |  |  |  |  |  |  | | | | |
|  | Not married | Reference | | | | | Reference | | | | |
|  | Married | 1.82 | <0.001 | | 1.58 | 2.09 | 1.94 | <0.001 | | 1.63 | 2.29 |
| Health status | |  |  |  |  |  |  | | | | |
|  | Poor | Reference | | | | | Reference | | | | |
|  | Fair | 0.52 | <0.001 | | 0.40 | 0.69 | 0.40 | <0.001 | | 0.27 | 0.57 |
|  | Good | 0.77 | 0.032 | | 0.60 | 0.98 | 0.71 | 0.034 | | 0.52 | 0.97 |
|  | Very good | 0.95 | 0.644 | | 0.75 | 1.19 | 0.87 | 0.347 | | 0.64 | 1.16 |
|  | Excellent | 1.18 | 0.141 | | 0.95 | 1.47 | 1.21 | 0.187 | | 0.91 | 1.62 |
| Survey year | |  |  |  |  |  | Reference | | | | |
|  | Year 2004 | Reference | | | | |  |  |  |  |  |
|  | Year 2008 | 1.06 | 0.419 | | 0.92 | 1.21 | 0.98 | 0.795 | | 0.87 | 1.11 |
|  | Year 2012 | 0.79 | 0.005 | | 0.67 | 0.93 | 0.63 | <0.001 | | 0.56 | 0.71 |
|  | Year 2016 | 0.53 | <0.001 | | 0.43 | 0.65 | 0.38 | <0.001 | | 0.33 | 0.43 |
| ^a^ PA: physical activity based on the PA Index, a score of 21 and above indicates active whereas a score lower than 21 indicates inactive. | | | | | | | | | | | |

**4. Association between physical activity (PA) and mammography screening utilization, stratified by race/ethnicity**

Table S4. Odds ratios and 95% confidence intervals from mixed effect logistic regressions of mammography based on PA among White women

| Variable | | Odds Ratio | | | *P* | 95% Conf. Interval | |
| --- | --- | --- | --- | --- | --- | --- | --- |
| PA ^a^ | | Lag PA | | | | | |
|  | Not active | Reference | | | | | |
|  | Active | 1.31 | 0.002 | | | 1.10 | 1.56 |
| Age | | 0.97 | 0.002 | | | 0.95 | 0.99 |
| Education | |  |  | |  |  |  |
|  | Less than high school | Reference | | | | | |
|  | High school/some college | 1.33 | 0.074 | | | 0.97 | 1.81 |
|  | College and above | 1.83 | 0.003 | | | 1.22 | 2.74 |
| Household income quintiles | |  |  | |  |  |  |
|  | $0 - $10,000 | Reference | | | | | |
|  | $10,000 - $18,000 | 1.47 | 0.003 | | | 1.14 | 1.90 |
|  | $18,000 - $29,000 | 2.00 | <0.001 | | | 1.52 | 2.64 |
|  | $29,000 - $49,000 | 2.36 | <0.001 | | | 1.76 | 3.16 |
|  | $49,000+ | 2.73 | <0.001 | | | 1.98 | 3.77 |
| Health insurance | |  |  | |  |  |  |
|  | Uninsured | Reference | | | | | |
|  | Insured | 5.68 | <0.001 | | | 3.04 | 10.59 |
| Region | |  |  | |  |  |  |
|  | Northeast | Reference | | | | | |
|  | Midwest | 0.99 | 0.956 | | | 0.71 | 1.38 |
|  | South | 1.03 | 0.828 | | | 0.76 | 1.41 |
|  | West | 0.97 | 0.914 | | | 0.91 | 1.42 |
| Marital status | | 0.14 |  | 0.102 | | 0.10 | 1.48 |
|  | Not married | Reference | | | | | |
|  | Married | 2.04 | <0.001 | | | 1.66 | 2.50 |
| Health status | |  |  | |  |  |  |
|  | Poor | Reference | | | | | |
|  | Fair | 0.36 | <0.001 | | | 0.22 | 0.55 |
|  | Good | 0.62 | 0.011 | | | 0.42 | 0.89 |
|  | Very good | 0.95 | 0.762 | | | 0.67 | 1.33 |
|  | Excellent | 1.30 | 0.125 | | | 0.93 | 1.82 |
| ^a^ PA: physical activity based on the PA Index, a score of 21 and above indicates active whereas a score lower than 21 indicates inactive. | | | | | | | |

| Table S5. Odds ratios and 95% confidence intervals from mixed effect logistic regressions of mammography screening based on PA among Black women | | | | | | |
| --- | --- | --- | --- | --- | --- | --- |
| Variable | | Odds Ratio | | *P* | 95% Conf. Interval | |
| PA ^a^ | | Lag PA | | | | |
|  | Not active | Reference | | | | |
|  | Active | 1.43 | 0.059 | | 0.98 | 2.09 |
| Age | | 0.94 | 0.002 | | 0.91 | 0.98 |
| Education | |  |  |  |  |  |
|  | Less than high school | Reference | | | | |
|  | High school/some college | 1.29 | 0.257 | | 0.83 | 2.01 |
|  | College and above | 1.54 | 0.229 | | 0.76 | 3.11 |
| Household income quintiles | |  |  |  |  |  |
|  | $0 - $10,000 | Reference | | | | |
|  | $10,000 - $18,000 | 1.60 | 0.33 | | 1.04 | 2.47 |
|  | $18,000 - $29,000 | 1.15 | 0.610 | | 0.68 | 1.94 |
|  | $29,000 - $49,000 | 1.58 | 0.156 | | 0.84 | 2.96 |
|  | $49,000+ | 1.24 | 0.564 | | 0.60 | 2.58 |
| Health insurance | |  |  |  |  |  |
|  | Uninsured | Reference | | | | |
|  | Insured | 17.23 | <0.001 | | 6.33 | 46.85 |
| Region | |  |  |  |  |  |
|  | Northeast | Reference | | | | |
|  | Midwest | 1.60 | 0.169 | | 0.81 | 3.14 |
|  | South | 1.01 | 0.960 | | 0.59 | 1.75 |
|  | West | 1.02 | 0.969 | | 0.41 | 2.52 |
| Marital status | |  |  |  |  |  |
|  | Not married | Reference | | | | |
|  | Married | 1.59 | 0.036 | | 1.03 | 2.45 |
| Health status | |  |  |  |  |  |
|  | Poor | Reference | | | | |
|  | Fair | 0.53 | 0.206 | | 0.20 | 1.41 |
|  | Good | 0.86 | 0.746 | | 0.35 | 2.11 |
|  | Very good | 0.74 | 0.510 | | 0.31 | 1.79 |
|  | Excellent | 0.80 | 0.630 | | 0.32 | 1.98 |
| ^a^ PA: physical activity based on the PA Index, a score of 21 and above indicates active whereas a score lower than 21 indicates inactive. | | | | | | |

| Table S6. Odds ratios and 95% confidence intervals from mixed effect logistic regressions of mammography based on PA among Hispanic women | | | | | | | |
| --- | --- | --- | --- | --- | --- | --- | --- |
| Variable | | Odds Ratio | | | *P* | 95% Conf. Interval | |
| PA ^a^ | | Lag PA | | | | | |
|  | Not active | Reference | | | | | |
|  | Active | 1.08 | 0.784 | | | 0.71 | 1.57 |
| Age | | 0.96 | 0.045 | | | 0.92 | 0.99 |
| Education | |  |  | |  |  |  |
|  | Less than high school | Reference | | | | | |
|  | High school/some college | 1.18 | 0.526 | | | 0.71 | 1.94 |
|  | College and above | 0.62 | 0.465 | | | 0.17 | 2.21 |
| Household income quintiles | |  |  | |  |  |  |
|  | $0 - $10,000 | Reference | | | | | |
|  | $10,000 - $18,000 | 0.90 | 0.695 | | | 0.55 | 1.48 |
|  | $18,000 - $29,000 | 0.87 | 0.667 | | | 0.45 | 1.66 |
|  | $29,000 - $49,000 | 1.08 | 0.855 | | | 0.48 | 2.41 |
|  | $49,000+ | 1.44 | 0.500 | | | 0.50 | 4.16 |
| Health insurance | |  |  | |  |  |  |
|  | Uninsured | Reference | | | | | |
|  | Insured | 2.50 | 0.006 | | | 1.29 | 4.83 |
| Region | |  |  | |  |  |  |
|  | Northeast | Reference | | | | | |
|  | Midwest | 0.61 | 0.459 | | | 0.59 | 3.63 |
|  | South | 0.37 | 0.020 | | | 0.38 | 1.87 |
|  | West | 0.35 | 0.015 | | | 0.58 | 2.81 |
| Marital status | | 0.18 |  | 0.025 | | 0.25 | 1.41 |
|  | Not married | Reference | | | | | |
|  | Married | 1.54 | 0.048 | | | 1.16 | 2.37 |
| Health status | |  |  | |  |  |  |
|  | Poor | Reference | | | | | |
|  | Fair | 0.59 | 0.239 | | | 0.25 | 1.41 |
|  | Good | 1.28 | 0.537 | | | 0.58 | 2.80 |
|  | Very good | 0.84 | 0.677 | | | 0.38 | 1.66 |
|  | Excellent | 1.47 | 0.401 | | | 0.60 | 3.63 |
| ^a^ PA: physical activity based on the PA Index, a score of 21 and above indicates active whereas a score lower than 21 indicates inactive. | | | | | | | |

**6. Effect modification by race/ethnicity on the association between PA and mammography screening utilization**

| Table S7. Coefficients (log of odds ratio) and 95% confidence intervals from mixed effect logistic regressions of MS utilization based on PA; effect modified by race/ethnicity (n= 18,157) | | | | |
| --- | --- | --- | --- | --- |
| MS Utilization | Coefficient  (95% Conf. Interval) | | | *P* |
| Current PA ^a^  Inactive  Active  Race/ethnicity  NH White ^b^  Hispanic  NH Black ^c^  Effect modification by race  NH White ^b^  Hispanic  Active vs Inactive  NH Black ^c^  Active vs Inactive | | Reference | | |
|  |  | 1.94 (1.70, 2.21) | <0.001 | |
|  |  | Reference | | |
|  |  | 0.97 (0.75, 1.26) | 0.824 | |
|  |  | 1.41 (1.14, 1.74) | 0.001 | |
|  |  | Reference | | |
|  |  | 0.58 (0.40, 0.84) 0.003 | | |
|  |  | 0.54 (0.39, 0.74) | <0.001 | |
| ^a^ PA: physical activity based on the PA Index, a score of 21 and above indicates active whereas a score lower than 21 indicates inactive. | | | | |

| Table S9. Percentages of household income quantiles based on race/ethnicity for women 40+ | | | | | | | | |
| --- | --- | --- | --- | --- | --- | --- | --- | --- |
|  |  | | Household income quantiles | | | | |  |
|  | $0 - $10,000 | $10,000 - $18,000 | | $18,000 - $29,000 | $29,000 - $49,000 | $49,000+ | Total | *P value ^a^* |
| Race/ethnicity |  | | | | |  |  | <0.001 |
| NH White ^b^ | 17.74 | 21.24 | | 21.06 | 20.47 | 19.49 | 100 |  |
| NH Black ^c^ | 47.35 | 20.67 | | 13.20 | 10.96 | 7.83 | 100 |  |
| Hispanic | 58.90 | 19.07 | | 10.61 | 6.96 | 4.46 | 100 |  |
| Total | 26.74 | 20.93 | | 18.72 | 17.55 | 16.07 | 100 |  |
| ^a^ *P* value based on Chi2 test. ^b^ NH White: Non-Hispanic White. ^c^ NH Black: Non-Hispanic Black. | | | | | | | |  |

**7. Cross tabulation of women’s races/ethnicities based on education and household income**

| Table S8. Percentages of education based on race/ethnicity for women 40+ | | | | | |
| --- | --- | --- | --- | --- | --- |
|  | Education | | | |  |
|  | Less than high school | High school/ some college | College degree or above | Total | *P value ^a^* |
| Race/ethnicity |  | | |  | <0.001 |
| NH White ^b^ | 14.63 | 67.36 | 18.01 | 100 |  |
| NH Black ^c^ | 34.26 | 53.41 | 12.33 | 100 |  |
| Hispanic | 62.87 | 33.76 | 3.37 | 100 |  |
| Total | 22.74 | 61.66 | 15.60 | 100 |  |
| ^a^ *P* value based on Chi2 test. ^b^ NH White: Non-Hispanic White. ^c^ NH Black: Non-Hispanic Black. | | | | |  |

**8. The pattern of mammography screening utilization by physical activity over the years**


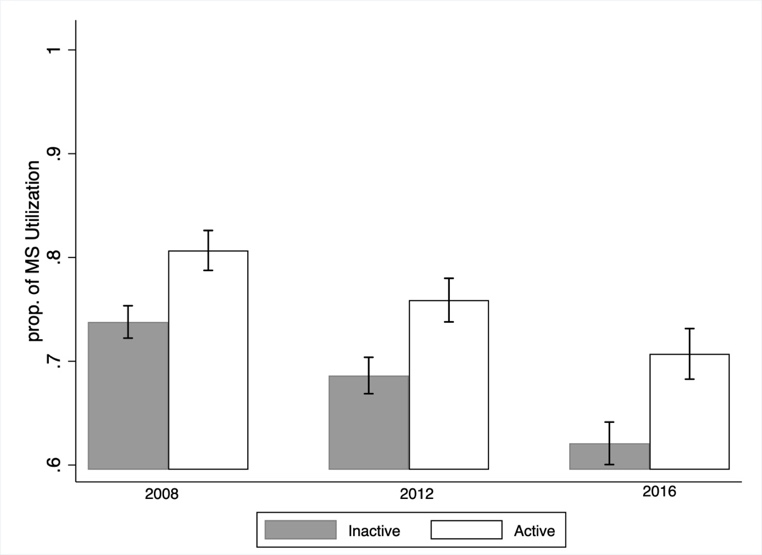


Figure S1. Proportion of Mammography Screening Utilization between Active and Inactive Women over the Years
